# Skeletal muscle contractility, self-reported pain and tissue sensitivity in females with neck/shoulder pain and upper Trapezius myofascial trigger points– a randomized intervention study

**DOI:** 10.1186/2045-709X-20-36

**Published:** 2012-11-25

**Authors:** Corrie Myburgh, Jan Hartvigsen, Per Aagaard, Anders Holsgaard-Larsen

**Affiliations:** 1 Institute of Sport Science and Clinical Biomechanics, University of Southern Denmark, Fyn, Odense 5230, Denmark; 2 Nordic Institute for Chiropractic and Clinical Biomechanics, Fyn, Odense, 5230, Denmark; 3 Department of Orthopeadic Surgery Odense University Hospital, University of Southern Denmark, Fyn, Odense 5230, Denmark

**Keywords:** Myofascial pain, Trigger points, Neck/shoulder pain, Mechanical outcomes

## Abstract

**Background:**

In relation to Myofascial Triggerpoints (MFTrPs) of the upper Trapezius, this study explored muscle contractility characteristics, the occurrence of post-intervention muscle soreness and the effect of dry needling on muscle contractile characteristics and clinical outcomes.

**Methods:**

Seventy-seven female office workers (25-46yrs) with and without neck/shoulder pain were observed with respect to self-reported pain (NRS-101), pressure-pain threshold (PPT), maximum voluntary contraction (F_max_) and rate of force development (RFD) at baseline (pre-intervention), immediately post-intervention and 48 hours post-intervention. Symptomatic and asymptomatic participant groups were each randomized into two treatment sub-groups (superficial (SDN) and deep dry needling (DDN)) after baseline testing. At 48 hours post-intervention participants were asked whether delayed onset muscle soreness (DOMS) and/or post-needling soreness had developed.

**Results:**

Muscle contractile characteristics did not differ between groups at baseline. Forty-six individuals developed muscle soreness (39 from mechanical testing and seven from needling). No inter-group differences were observed post-intervention for F_max_ or RFD for the four sub-groups. Over the observation period, symptomatic participants reported less pain from both SDN (p= 0.003) and DDN (p=0.011). However, PPT levels were reduced for all participants (p=0.029). Those reporting DOMS experienced significant decreases in PPT, irrespective of symptom state or intervention (p=0.001).

**Conclusions:**

In selected female neck/shoulder pain sufferers, maximum voluntary contraction and rapid force generation of the upper Trapezius was not influenced by clinically relevant self-reported pain or the presence of diagnostically relevant MFTrPs. Dry needling, deep or superficial, did not affect measured functional outcomes over the 48-hour observation period. DOMS affected participants uniformly irrespective of pain, MFTrP status or intervention type and therefore is like to act as a modifier.

**Trial registration:**

Clinical Trials.gov- NCT01710735

**Significance and Innovations:**

The present investigation is one of the first to examine the hypothesis of gross muscle contractile inhibition due to the presence of diagnostically relevant MFTrPs.

Individuals suffering from clinically relevant levels of self-reported pain are able to tolerate maximum voluntary contraction testing, but delayed onset muscle soreness (DOMS) is a likely side-effect irrespective of symptom status. As a consequence, its confounding effect during subsequent testing must be taken into account.

## Background

Musculoskeletal problems of the neck/shoulder region are a growing 21^st^ century health care concern [1]. The prevalence of long standing musculoskeletal illness in Denmark is estimated at around 13% and approximately 20% of the adult Danish population has experienced pain in the neck region during the previous month [2]. Furthermore, neck/shoulder pain more commonly affects women than men [1].

The study, identification and treatment of hyperirritable skeletal muscle fibers, known as myofascial trigger points (MFTrPs or TrPs) have emerged as an important category within the area of general musculoskeletal pain [3-5]. Epidemiological data specific to Myofascial Pain Syndrome (MPS) is still somewhat unclear, but the mean prevalence of the condition among adults aged 30 to 60 years is estimated at 37% for men and 65% for women [6].

MFTrPs are typically identified through a combination of manual palpation and patient feedback [7,8]. During this process, referred to as global assessment (GA), the examiner establishes whether presenting symptoms such as local and referred pain, match the location and patterns of referral known to exist in relation to TrPs of a particular muscle. GA has been found to be a valid and reliable manner in which to establish the diagnostic relevance of MFTrPs (also referred to as an ‘active’ TrP) [7]. A TrP that is not associated with the presenting clinical picture can be termed ‘latent’.

An important issue, which continues to hamper the development of the understanding of MFTrP phenomena, relates to a relative dearth in measurable outcomes demonstrating pathophysiology. For example, it has been argued that that skeletal myofibers may fatigue prematurely and may be weakened due to autogenic reflex inhibition of the tissues containing MFTrP(s) [9,10]. Although plausible, this theory remains essentially unsubstantiated and robust measures are required to observe the mechanical and neuromuscular behavior of affected musculature [11]. Thus, as an initial step to fully understand what might be expected with respect to gross muscle contractility, an assessment of the muscles mechanical properties is required.

In the exercise and occupational health sciences, the maximum voluntary contraction force (F_max_), and the capacity for rapid force generation, so-called rate of force development (RFD), are routinely used to quantify mechanical and neuromuscular parameters in intact human skeletal muscle [12-15]. Studies have demonstrated reduced F_max_ and RFD among females with chronic neck muscle pain compared to asymptomatic controls [14,15]. However, these parameters do not seem to have been evaluated in the MFTrP context. An aspect that perhaps limits the use of maximum voluntary contraction characteristics is the propensity of test subjects to develop delayed onset of muscle soreness (DOMS) [16-18]. This well-known phenomenon is commonly experienced within 24 hours after unaccustomed exercise, typically lasting for 1 to 4 days. DOMS typically is characterized by a painful sensation when contracting, stretching, or exerting pressure on the stressed muscle. Consequently, self-limiting increased tissue sensitivity, pain and decreases in maximum muscle force have been observed in relation to DOMS [18].

As an intervention, dry needling holds promise for the clinical resolution of MFTrPs implicated in MPS [19,20]. Two sub-types, superficial (SDN) and deep dry needling (DDN) techniques are routinely applied in the clinical setting. SDN is thought to achieve its effect indirectly, inhibiting C fiber pain impulses, where as DDN stimulates affected muscle directly, causing rapid depolarization of affected fibers in the area of the MFTrP nidus. DDN appears to affect clinical outcomes more strongly, but also gives rise to post-needle soreness; a side effect stemming from the repeated insertions, required to abolish local twitch responses (LTR) [20,21].

To start exploring the viability of muscle testing as an outcome measure in relation to MFTrPs, the present study intended to determine whether contractile muscle characteristics are affected by clinically relevant MFTrPs, whether post-intervention muscle soreness and DOMS affects clinical and/or mechanical outcomes and whether muscle characteristics (F_max_ and RFD) and clinical outcomes are affected by dry needling.

The specific objectives study were (i) whether F_max_ and/or RFD differ significantly between symptomatic and asymptomatic individuals, (ii) if post-testing DOMS was reported in relation to symptom status and/or depth of needling, (iii) if post-needling soreness occurred in relation to symptom status and/or depth of needling (iv) whether dry needling of a clinically relevant MFTrP influences self-reported pain, tissue sensitivity and maximum voluntary contraction and (v) whether DOMS is likely to act as a modifier.

## Method

### Design

A randomized, intervention study conducted in a human performance laboratory.

### Study population

Through local newspaper adverts responses were solicited from females aged 20 and 46 years, performing office work for four hours or more a day. These criteria were aimed at limiting age related variation in strength reductions and MFTrP prevalence [22]. It is unclear whether occupational mechanical overload (either acute or chronic) is associated with elevated prevalence of MFTrPs, however, in order to reduce heterogeneity we sampled participants sharing a common risk factor [23]. A drawing, corresponding with the area covered by the upper Trapezius muscle was provided to clarify our study target area. Both individuals suffering from neck/shoulder pain and pain free persons were encouraged to respond in order to create a case mix. This enabled us to create four sub-groups (two asymptomatic and two symptomatic). A project administrator screened respondents telephonically.

Respondents were excluded if they presented with a history of chronic, systemic pathology (e.g. hemophilia), pre-existing neck/shoulder pathology/surgical procedures, suffered clinical depression, were involved in health-related legal action, were likely to be pregnant, used anti-inflammatory and/or chronic pain medication, had received dry needling for a shoulder/neck disorder within 6 months prior to the study (to raise the level of patient naiveté), suffered from needle phobia or had a body-mass index (BMI) ≥ 31. The BMI criterion was aimed at reducing sample heterogeneity due to large variation between soft tissues present over the shoulder area.

After agreeing to participate, an information letter and background questionnaire was posted to the subjects electronically. The questionnaire was adapted from similar musculoskeletal investigations conducted in the workplace context [24]. A consultation date within 5 days of the telephonic contact was then scheduled.

### Clinical evaluation

Subjects presented to the Institute of Sport Science and Clinical Biomechanics, University of Southern Denmark. An evaluation was conducted by an experienced musculoskeletal clinician (CM) with 15 years exposure to MFTrP evaluation, who confirmed general study eligibility inclusion/exclusion criteria, determined symptomatic/asymptomatic status and MFTrP status, gathered anthropometrical data and randomized participants.

Our operational definition of neck and/shoulder pain was pain in the region of the upper Trapezius muscles, therefore only participants satisfying this criteria could qualify as symptomatic for the purposes of this study [9]. A cut-point was established that defined a participant with a score ≥3 on the NRS-101, as a symptomatic subject [25]. This criterion was introduced in order to exclude participants presenting with clinically irrelevant levels of self-reported pain.

The diagnostic relevance of MFTrPs was established by means of clinician global assessment. GA is both a reliable method for evaluating MFTrPs and allows for the distinction between so-called ‘active and latent’ TPs [7]. Furthermore, participants found to have clinically relevant MFTrPs capable of referring pain overlapping with that of the upper Trapezius musculature were excluded from the study, these included: the cervical Multifidi, Splenius cervicis, Levator Scapulae, Supraspinatus, Infraspinatus and Scalenes [9]. The distinction between symptomatic and asymptomatic individuals in this study therefore rested on the intensity of self-reported pain and the presence of a diagnostically relevant (active) MFTrP in the upper Trapezius muscle.

Our specific criteria for the symptomatic group therefore were: a clinically relevant MFTrP of the upper Trapezius musculature and self- reported pain of ≥3 on an eleven-point numerical pain rating scale (NRS-101). Our specific criteria for the asymptomatic group were: absence of a clinically relevant MFTrP of the upper Trapezius musculature and self-reported pain of 0. Respondents who did not meet the specific criteria for either group were excluded.

### Randomisation

Participants were allocated into particular sub-groups, using computer generated random numbers tables. Two tables were generated, one for symptomatics, the other for asymptomatics. Each contained the numbers 1-50, allocated prior to the study to a 0 (DDN) or 1(SDN) group. As participants were inducted, their participant number would thus randomly correspond to a group.

### Evaluation of mechanical muscle function

**Figure 1:**
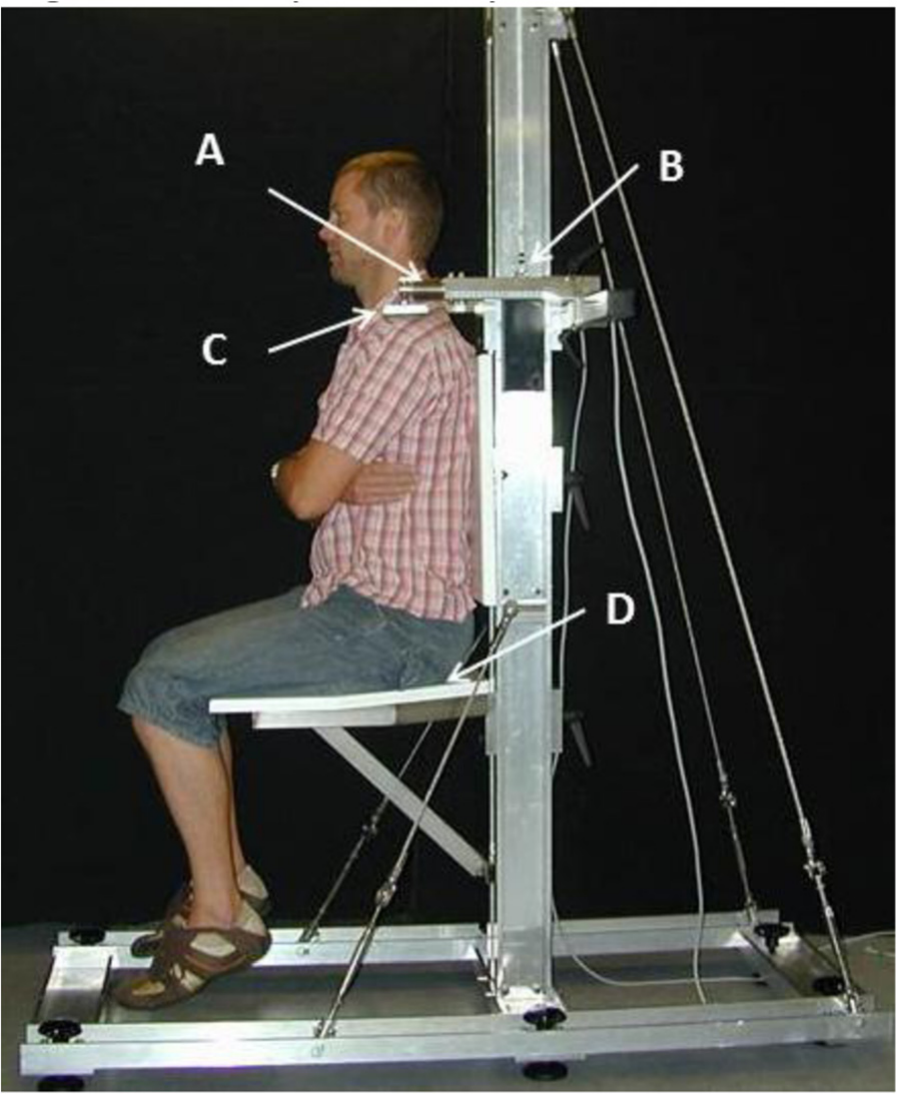
**Trapezius Dynamometer.** Trapezius Dynamometer in the shoulder elevation test position. The dynamometer consists of **A**) Strain Gauge force transducers, **B**) Adjustable slide rail to individualize height, **C**) Shoulder pad, and **D**) Adjustable seat to ensure no contact between feet and ground.

Measurements of maximal isometric muscle force (F_max_), and rate of force development (RDF), were performed for the shoulder elevators (shoulder joint positioned in an anatomic neutral position and elbow extended) and shoulder abductors (shoulder joint positioned in an anatomic neutral position and elbow joint in 90^o^ of flexion) (Figure 1). Subjects were seated and strapped in a custom-built isometric shoulder elevation/abduction dynamometer chair based upon strain-gauge technology, connected to a computer with custom developed software (MathWorks, MatLab) [15]. The strain-gauge signals were immediately converted into Newton (N), and stored digitally for further analysis. The chair was adjusted to allow for differences in individual anthropometrics (i.e. chair height, where the subjects feet were not allowed to touch the ground, backrest height, shoulder height and shoulder distance). These adjustments were made for both the position of elevation test and then for the abduction test. Individual settings were noted and used at every test occasion. F_max_ and RFD measured in the initial 200ms from the instant of contraction onset (RFD_200 ms_) were determined for the shoulder elevators and abductors (intervention side only) in all four sub-groups. Instant of contraction onset was operationalized as the first observable deviation of the signal from baseline. Shoulder elevation was tested first, followed directly by the abduction test directly after. For both shoulder elevation and abduction, subjects were instructed to contract as explosively and forcefully as possible. Verbal encouragement during testing and online visual feedback of the instantaneous dynamometer force on a computer screen was provided. Contraction duration was 6 seconds and successive contractions were performed ad libitum until no further increase in F_max_ could be observed. F_max_ was measured for each contraction and the contraction with highest F_max_ from each test was selected for further analysis. Mechanical muscle data were recorded at baseline, immediately post needling and 48 hours after dry needle insertion treatment. The test-procedure was performed bilaterally but only unilateral data from the affected side (for symptomatic) or one side randomly chosen (for asymptomatic) was used. Before tests, participants performed a standardized warm up routine for the neck and shoulders.

### Pain-related outcome measures

Self-reported pain (NRS-101 eleven point pain rating scale) and pressure-pain threshold (PPT) algometry are commonly employed as clinical outcomes in MPS research to observe subjective pain experience and tissue sensitivity, respectively [26,27]. The former parameter was recorded at baseline and at 48 hours post-intervention, whilst the latter was recorded at baseline, immediately post-intervention and 48 hours post-intervention. PPT measures were obtained from MFTrP sites in symptomatic participants or typical Trapezius MFTrP location ‘reference sites’ in asymptomatic participants [9]. Specific sampling sites were marked with permanent ink during evaluation and covered with a waterproof plaster. Participants were also asked not to wash or touch the area if possible. All PPT values were recorded in kilopascal.

### Post-needling soreness and post-testing DOMS

At 48 hours post-intervention evaluation, we asked participants: *Did you experience an increase in muscle discomfort and/or soreness starting the day after the first examination?* In cases where soreness had developed, we further discerned whether participants felt the soreness due to needling (unilateral in the proximity of needle insertion), mechanical testing (diffuse and invariably noted bilaterally) or both.

### Intervention procedures

Dry needle insertion took place immediately after baseline measurement. A 3” (25mm) acupuncture needle was used in all needle procedures (Cloud and Dragon, Shanghai Xinhua E-General Merchandise Co., Ltd). MFTrP stimulation occurred on the side of which the subject showed hand dominance, in the case of bilateral findings. Interventions were either in the form DDN [28] or SDN insertion [20]. Participants were needled in a seated position, making use of a supported treatment chair (Chattanooga Adapta® MC-100 Portable Massage Chair). In all instances, a single dose of needling was administered immediately after the baseline mechanical muscle evaluation. A repeated insertion, clockwise fanning motion was used. Fanning is thought to result in more favourable outcomes than single insertion protocol, due to a higher likelyhood inducing hyperstimulation analgesia and exhausting the MFTrP twitch response [26].

In both symptomatic and asymptomatic (control) subjects the needle depth was marked post-intervention and the needle depth recorded with calipers (Mitutoyo absolute model no.cd-20cpx).

### Deep dry needling

DDN of symptomatic participants was based on the appearance and exhaustion of the twitch response phenomenon [29]. The twitch response is a reliable criterion for the presence of trigger points when fibers in the immediate vicinity of the MFTrP nidus are stimulated [30,31]. The purpose of needling in this sub-group therefore was to elicit and exhaust twitch responses with repeated fanning needling insertion [32]. An experimental pilot protocol was developed on five non-participants (with appropriate anthropometric characteristics), in order to develop an idea of the needle penetration that might be expected as well as the time that would be required. We observed that, in order to repeatedly elicit twitch responses, no less than 10mm of the needle had to penetrate the epidermis over a period of 90 seconds.

As no twitch response would exist for asymptomatic subjects, none could be induced. Therefore, the needles were marked at 10mm pre-intervention, so that the insertion depth would be similar to participants receiving deep needle insertion.

### Superficial dry needling

We standardised this procedure, by tapping (using the index finger) the needle into the epidermis until it remained erect and could support its own weight. Again we piloted this protocol on 5 non-participants to determine what depth of penetration this resulted in. The needle penetration depth was determined to be 5mm. Current guidelines for superficial needling concurred with our findings, suggesting an insertion depth of between 5-10mm [20]. No twitch response was expected for symptomatic or asymptomatic subjects, therefore intervention time was standardised to 90 seconds.

### Blinding

Both participants and mechanical evaluation technicians were blinded with respect to the type of intervention applied, i.e. superficial or deep needle insertion.

### Statistical analysis

We estimated that optimal sub-group sizes for this study would require data from 31 participants. The calculation was based on detecting differences of 25% in self-reported pain after treatment post-intervention, a 2-tailed test, α=0.05 and a power of 80%. Normal distribution for the dataset was not assumed, thus all evaluations were tested using non-parametric statistics. Inter-group comparison of symptomatic and asymptomatic subjects at baseline was conducted using Mann–Whitney U tests for all outcomes observed. The frequency of the development of post-intervention soreness was recorded and compared between participant groups using the Mann–Whitney *U* test. To analyse intra-group effects over time we conducted firstly a Friedman’s test, in order to observe whether median values in at least one group changed significantly with respect to any of the others. Furthermore, the Wilcoxon’s signed rank test was performed in order to observe whether significant median value changes occurred within sub-groups over time. Finally, inter-group differences immediately after and 48 hours post-intervention were analysed, again by means of the Mann–Whitney *U* test. PPT, F_max_ and RFD comparisons where conducted at three time points, namely baseline (pre-intervention), immediately post-intervention and at 48 hours post-intervention, whereas self-reported pain (NRS-101) comparisons were conducted only pre- and 48 hours post-intervention. To determine whether age or BMI was significantly correlated with self-reported pain, tissue sensitivity or mechanical parameters observed, Spearman’s correlation testing was performed. We also used a Bonferroni correction to adjust for multiple comparisons.

Means, medians, SDs and 95% CIs were calculated for all dependent variables. We also calculated box plots to compare the onset and development of DOMS. Statistical significance was set at *p≤0.05.* All muscle strength/RFD outcome measures were normalized relative to the participant’s bodyweight in kilograms (N/kg). Data were exported to STATA v11 (http://www.stata.com) and IBM**®**SPSS v19 for statistical analysis.

Ethical clearance (S-20070094HJD) was granted through the local ethics committee (Region of Southern Denmark).

## Results

### Participants and baseline comparison

**Figure 2:**
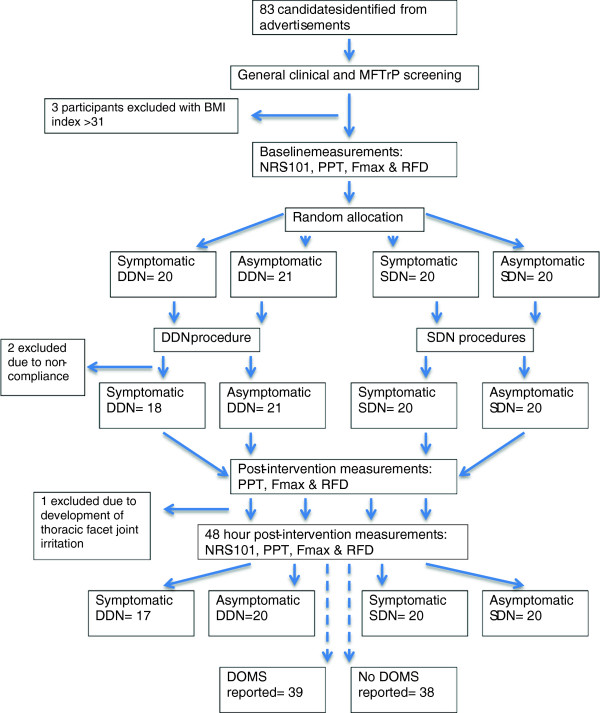
Flow of participants, data collection points and outcome measures.

As illustrated in Figure 2 eighty-three candidates agreed to the study conditions and signed a written inform consent. During the course of the study six participants were excluded; three were found to have a BMI of ≥ 31 (the participant BMI self-calculations were erroneous), two participants elected not to complete the protocol and one subject developed facet joint irritation in the mid thoracic spine. Symptomatic (n=37) and asymptomatic participants (n=40) were observed to be anthropometrically similar, however, the median age of symptomatic participants was 31 years, versus 24 in the asymptomatic comparison group (p= 0.007) (see also Table 1). Of the symptomatic subjects, 76% had consulted a health care practitioner in the preceding 12 months over symptoms in the neck/shoulder area, compared to 24% of asymptomatic participants.

**Table 1:** Baseline comparison (means) of symptomatic and asymptomatic participants

**All participants (N= 77)**	**Age**	**NRS-101**	**BMI**	**PPT**	**F**_**max**_**-shoulder abductors**	**F**_**max**_**- shoulder elevators**	**RFD- shoulder abductors**	**RFD- shoulder elevators**
Symptomatic (n=37)	46.07	58.35	40.09	38.55	36.49	33.20	35.00	33.69
Asymptomatic (n=40)	32.46	21.10	37.99	39.41	37.47	39.62	38.84	39.16
p-value	0.007	<0.0001	0.680	0.866	0.842	0.193	0.440	0.267

As expected, self-reported pain was significantly higher among the symptomatic group (median= 5 versus 0) (p<0.0001). No significant differences were observed at baseline between symptomatic and asymptomatic participants for PPT, F_max_ or RFD (Table 1).

### Onset of post-needling soreness and DOMS

At 48-hour post-intervention, 46 participants reported the development of shoulder muscle soreness (39 mechanical testing versus 7 needle insertion). Two symptomatic participants receiving DDN and one receiving SDN attributed their soreness to the combination of needling and mechanical testing. As indicated by Table 2, the proportion of participants reporting muscle soreness due to mechanical testing ranged between 45% and 57% and was unrelated to symptoms or needling procedure. By contrast, muscle soreness attributed to needle insertion was observed almost exclusively among symptomatic participants receiving DDN.

**Table 2:** Participant perceived soreness 48 hours post-intervention

	**All participants (N=77)**	**Sympt DDN**	**Sympt SDN**	**Asympt DDN**	**Asympt SDN**	**p- value**
Soreness from strength test	39 (50.6%)	8 (47.1%)	9 (45.0%)	12 (57.1%)	10 (52.6%)	0.867
Soreness from needle intervention	7 (9.1%)	5 (29.4%)	1 (5%)	0 (0%)	1 (5.3%)	0.010

DDN= deep dry needling, SDN= superficial dry needling.

### Effects of superficial versus deep dry needling

#### Needle insertion depths

The comparative needle insertion depth was 6,0mm (SD 1.2) for SDN and 12,3mm (SD 2.2) for DDN (p<0.001).

#### F_max_ and RFD

In the asymptomatic SDN group, F_max_ was decreased from baseline to post intervention (p=0.01), whilst RFD increased between the baseline and 48-hour follow-up assessment (p= 0.007). However, no other significant changes were observed from baseline to follow-up in the effect of SDN and DDN (See Additional file 1).

#### Self-reported pain

Both symptomatic intervention groups experienced a reduction in self-reported pain between baseline and follow-up (Table 3) and no significant difference between DDN and SDN was observed at 48 hours post-intervention (p-values remained significant at the 0.05 threshold after Bonferroni multiple test adjustment) see also Additional file 2. A statistically significant difference remained between symptomatic and asymptomatic DDN sub-groups at 48 hours (p<0.0001).

**Table 3:** Inter and intra-group comparison by symptom grouping

**NRS-101 Baseline**							**NRS-101 48-hour F/U**							
**Sub-group**	**mean**	**95% CI**		**median**	**95% CI**		**mean**	**95% CI**		**median**	**95% CI**		_**Within-group comparison (p-value)**_	_**Between-group comparison (p-value)**_
S DDN	5.59	4.68	6.50	5.00	4.00	7.00	3.41	2.31	4.52	4.00	2.01	4.99	0.011*	S DDN vs As DDN p<0.0001
S SDN	5.40	4.48	6.32	5.00	4.12	6.88	4.60	3.62	5.58	4.50	3.12	6.00	0.003*	
As DDN	0.24	0.00	0.48	0.00	0.00	0.00	0.71	0.10	1.33	0.00	0.00	0.55	0.041	
As SDN	0.26	0.00	0.53	0.00	0.00	0.00	0.26	0.00	0.65	0.00	0.00	0.00	0.040	

S= Symptomatic, As= Asymptomatic, DDN= deep dry needling, SDN= superficial dry needling.

*P-values remained significant at the 0.05 threshold after Bonferroni multiple test adjustment.

#### Pressure-pain threshold

PPT measures indicated that tissue sensitivity increased significantly for the entire cohort over time (p=0.029). However, DDN and SDN sub-group analysis indicated no significant changes within or between group changes immediately or 48 hours post-intervention see also Additional file 3.

### Effect of post-testing DOMS

#### F_max_ and RFD

In the sub-group reporting DOMS, F_max_ for shoulder abduction increased between baseline and follow-up (p=0.004). Maximum elevator strength was decreased immediately post-intervention, but returned to baseline values 48 hours post-intervention. Similarly, RFD for shoulder elevation initially fell below baseline values, but then recovered over the subsequent 48-hour interval (p≤0.0001). No significant inter-group comparison differences were observed for either of these parameters.

#### Self-reported pain

**Figure 3:**
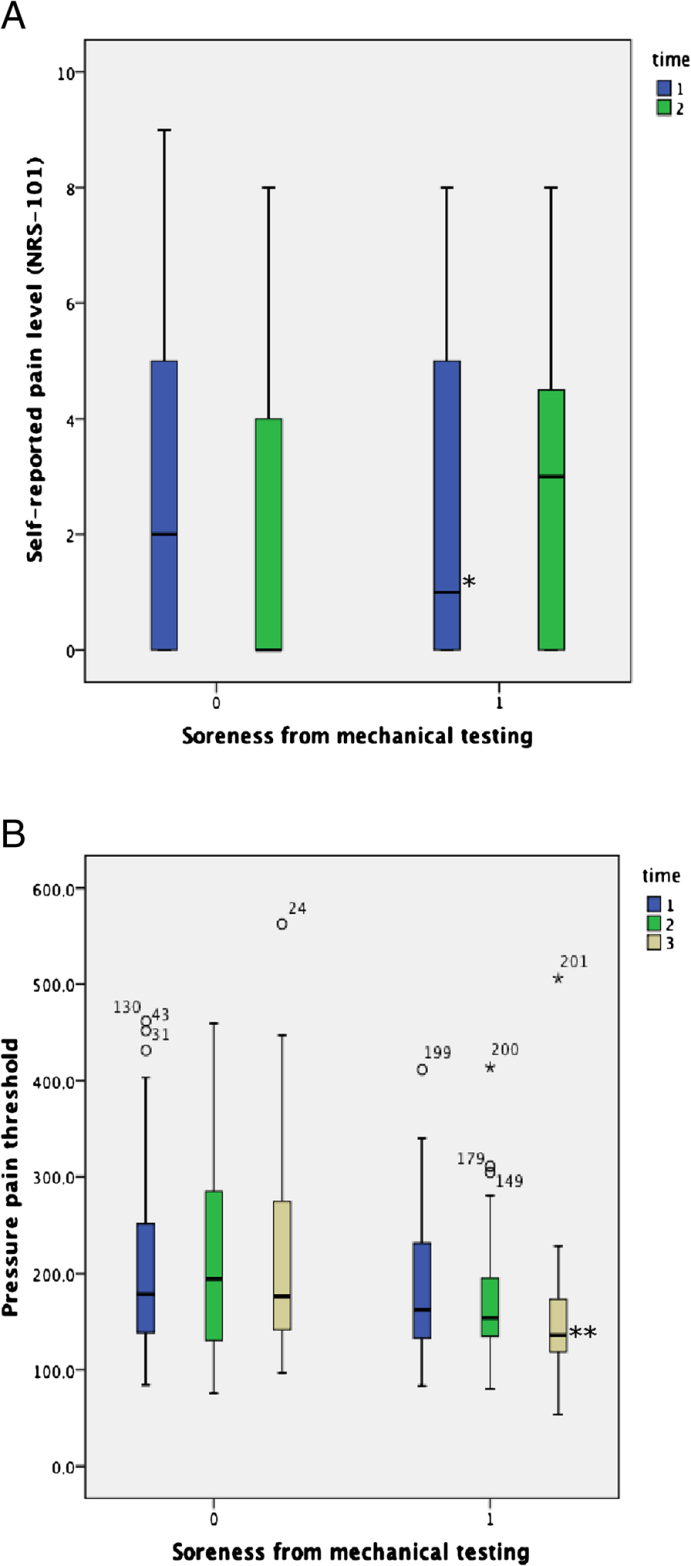
Self-reported pain (A) and pressure-pain thresholds (B) at baseline and after 48 hours in participants reporting the development of soreness versus those not reporting soreness.

A drop in self-reported pain was observed within the group unaffected by muscle soreness (p=0.005) (Figure 3A). In contrast, participants developing soreness reported increasing levels of self-reported pain. However, this observation did not reach statistical significance for the inter-group comparison 48 hours post-intervention.

#### Pressure-pain threshold

PPT among the participants reporting DOMS decreased between baseline and follow-up (median change score (MCS) -33.99) (p≤0.0001), but remained constant for those unaffected by DOMS (MCS 3.49). At 48-hours post-intervention, PPT was significantly depressed, indicating increased tissue sensitivity for participants experiencing DOMS (p=0.001) (see Figure 3B).

#### Correlation analysis

As shown in Table 4, co-efficients ranged between r= -0.449 (F_max_ shoulder elevators) and r=0.050 (RFD shoulder elevators), thus indicating no consistent relationship to exist between age and maximum shoulder elevation strength capacity.

**Table 4:** Spearman’s correlation of age by mechanical parameters

**N=77**	**F**_**max**_**- shoulder abductors**			**F**_**max**_**- shoulder elevators**			**RFD- shoulder abductors**			**RFD- shoulder elevators**			**Age**
**Interval**	**1**	**2**	**3**	**1**	**2**	**3**	**1**	**2**	**3**	**1**	**2**	**3**	
Age (CC)	-.102	-.160	-.170	-.285^*^	-.380^**^	-.449^**^	-.036	.001	-.107	.050	-.100	-.117	1.0
p-value	.382	.167	.151	.013	.001	<0.0001	.758	.996	.368	.671	.390	.327	.

Interval 1= baseline, interval 2= immediately post-intervention, interval 3= 48 hours post-intervention. **Correlation is significant at 0.01 (2-tailed), *Correlation is significant at 0.05 (2-tailed).

## Discussion

### Summary of findings

This study was, to the best of our knowledge, the first to apply maximum voluntary muscle contraction testing to the investigation of MFTrPs. Notably maximum contractile force (F_max_) and rapid force capacity (RFD) were unaffected by clinically relevant self-reported pain or the presence of diagnostically relevant MFTrPs. Dry needling at different depths, did not alter tissue sensitivity. DDN was somewhat more effective in reducing self-reported pain compared to SDN. Post-testing DOMS occurred commonly and was distributed uniformly among the cohort, where as post-needling soreness occurred rarely and almost exclusively among symptomatic participants receiving DDN. Both self-reported pain and tissue sensitivity were modified by the development of DOMS.

### Mechanical muscle function

No differences were observed for F_max_ or RFD in either of our analyses at baseline or over time. This indicates that neither symptom state, intervention type or post intervention soreness significantly impacted participant contractile characteristics. Thus, despite reporting elevated levels of self-reported pain and harbouring clinically relevant myofascial MFTrPs in the neck/shoulder area, symptomatic participants did not differ from asymptomatic counterparts with respect to maximum force capacity or rate of force development characteristics. Therefore, it appears that participants did not experience inhibition of gross muscle contractility, due to these factors [9].

### Onset of post-needling soreness and DOMS

Half the participants in this study, irrespective of symptom state, reported developing post-testing DOMS. Therefore it is likely that the increase in tissue sensitivity presently observed, was a reaction to the process, developing after the two doses of high intensity isometric muscle contractions elicited during the dynamometric evaluation.

The occurrence of post-needling soreness has been reported to range between 50 and 55%, depending on the needling procedure used [20,33]. It was therefore interesting to note a relative low occurrence rate of ≈30% in the DDN sub-group. Furthermore, post-needling soreness was rare amongst the remaining groups, despite the multiple needle insertions procedure used.

### Self-reported pain and tissue sensitivity

Dry needling (both DDN and SDN) elicited a reduction in self-reported pain in symptomatic participants. In contrast, asymptomatic subjects appeared to be developing pain. This was particularly noted in the case of the asymptomatic DDN sub-group. It is likely DOMS may have undermined observed beneficial effects in this outcome.

Finally, our results indicate that dry needling at different depths, did not alter tissue sensitivity either immediately after or at 48 hours post-intervention.

### Active control intervention

We argued that SDN appears to have clinical effects beyond that of a control/placebo intervention; the effect stemming from c-fibre inhibition, rather than direct stimulation of the TrP nidus. Furthermore, in designing the present study we took steps to ensure that any effect observed from SDN could not have been due to direct contact with muscle fibres. Thus, if both interventions induce an indirect effect and only DDN directly affected the myofascial tissues as indicated by the twitch response, then it stands to reason that SDN effectively performed the role of an active control in our study.

### Study strengths and limitations

The observed age difference between symptomatic and asymptomatic subjects may have affected the present results. However, this is unlikely given the inconsistent associations observed in the correlation analysis. Age is thought to have negative effect on MFTrP phenomena [34] and mechanical muscle function characteristics [22]; therefore it is unlikely that the symptomatic participants would have benefitted from skewness in age. Our symptom-based sub-group analysis was underpowered based on sample size calculation. Thus, type-II errors cannot be discounted. It is unclear from our results whether differences exist between SDN and DDN with respect to reducing self-reported pain as confounding due to DOMS, may have altered tissue sensitivity measurements after 48 hours. Our asymptomatic participants had also received previous treatment for their neck pain; it would therefore be prudent to determine neck pain status on more than self-reported pain at the point of inclusion. More clarity is required as to whether individuals are able to differentiate between soreness and DOMS. Establishing clinical relevance fell outside the scope of this study.

The present study methodology was innovative in its use of muscle contractility outcomes that accounted not only for maximal contractile strength (F_max_), but also for the ability to investigate the exertion of rapid muscle force characteristics (RFD). Interestingly, in this context neck/shoulder pain sufferers were willing and able to commit to three sessions of strenuous maximum voluntary contraction testing that were characterized by high levels of RFD, even after reporting the onset of DOMS. This finding appears to challenge the notion of painful inhibition and requires further study. It also appears that the onset of DOMS may result in an uncoupling of tissue sensitivity and self-reported pain in symptomatic individuals. In other words, participants with sore muscles may yet demonstrate reduced levels of subjective pain.

## Conclusions

Skeletal contractile muscle function did not differ between participants harbouring diagnostically relevant MFTrPs and without neck/shoulder pain versus participants who did not. This was apparent both at baseline and 48-hours after a mechanical intervention via dry needle insertion. DOMS was reported uniformly in response to muscle strength/RFD testing in half the participants. Dry needle stimulation of clinically relevant MFTRPs, both superficially or deeper into the skeletal muscle reduced self-reported pain in symptomatic subjects, apparently without affecting tissue sensitivity. DDN was somewhat more effective in this regard, but post-needle soreness in symptomatic participants was also higher (30% versus 5%). Post-intervention DOMS was induced uniformly among participants and is likely to have led to poorer pain-related outcomes being observed.

## Competing interests

We declare that no financial or other conflicts of interest exist with regard to this work.

## Authors' contributions

All authors read and approved the final manuscript.

## Supplementary Material

Additional file 1 Boxplot demonstrating Maximum Voluntary Contraction (MVC) for shoulder elevation for the four intervention sub-groups over time. Click here for file

Additional file 2 Boxplot demonstrating PPT levels for the intervention sub-groups over time. Click here for file

Additional file 3 Boxplot demonstrating self-reported pain levels for the intervention sub-groups over time. Click here for file
