# Supplementary material for: Skeletal muscle contractility, self-reported pain and tissue sensitivity in females with neck/shoulder pain and upper Trapezius myofascial trigger points– a randomized intervention study
Source: Chiropr Man Therap. 2012 Nov 25;20:36. doi: 10.1186/2045-709X-20-36 (PMC3599669; doi:10.1186/2045-709X-20-36)

Additional file 1: Boxplot demonstrating Maximum Voluntary Contraction (MVC) for shoulder elevation for the four intervention sub-groups over time.


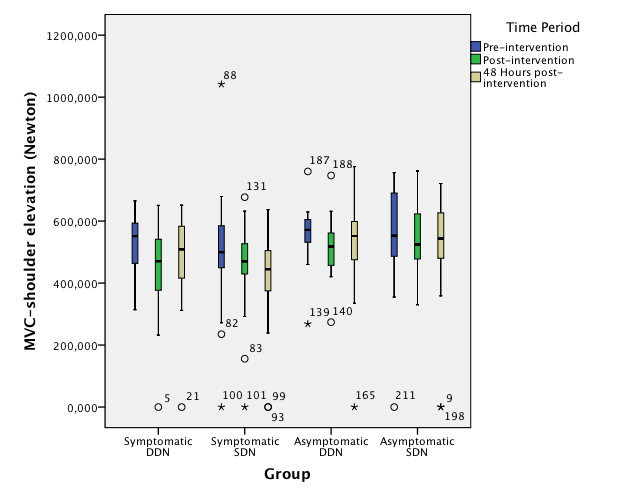


Boxplot demonstrating rate of force development (RFD) for abduction for shoulder elevation for the intervention sub-groups over time.


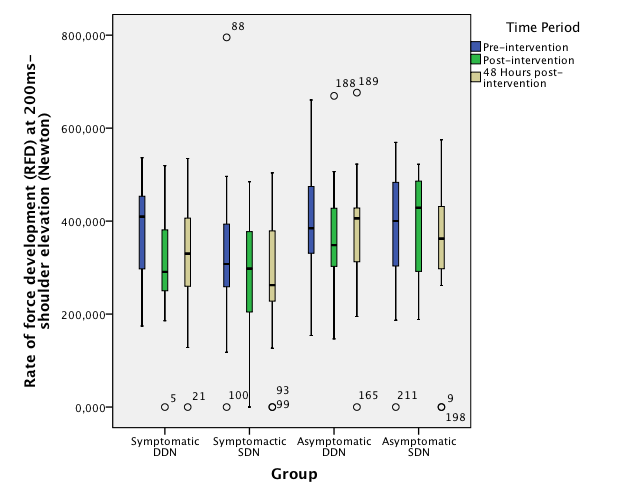

Supplement: Additional file 1 — Boxplot demonstrating Maximum Voluntary Contraction (MVC) for shoulder elevation for the four intervention sub-groups over time. [file 2045-709X-20-36-S1.docx]
