# Supplementary figures and images for: Skeletal muscle contractility, self-reported pain and tissue sensitivity in females with neck/shoulder pain and upper Trapezius myofascial trigger points– a randomized intervention study
Source: Chiropr Man Therap. 2012 Nov 25;20:36. doi: 10.1186/2045-709X-20-36 (PMC3599669; doi:10.1186/2045-709X-20-36)

Additional file 2: Boxplot demonstrating PPT levels for the intervention sub-groups over time.


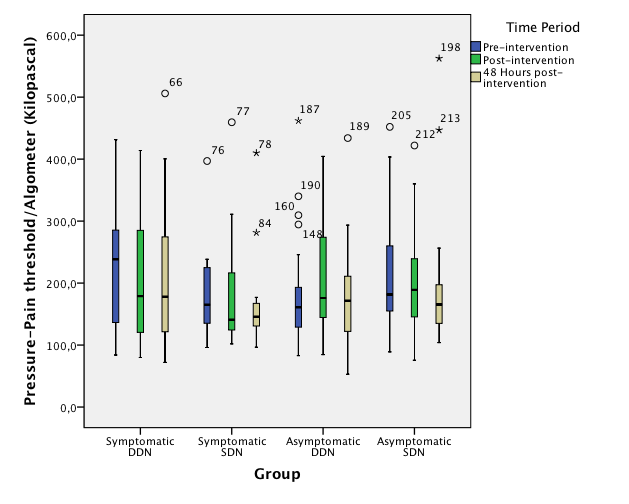

Supplement: Additional file 2 — Boxplot demonstrating PPT levels for the intervention sub-groups over time. [file 2045-709X-20-36-S2.docx]
